# Dr. Clough on an Extraordinary Twin Case

**Published:** 1811-01

**Authors:** H. G. Clough

**Affiliations:** 68, Berner's-street


*To the Editors of the Medical and Physical Journal.*

GENTLEMEN,

As nothing tends more towards the elucidation of medical science than the records of cases and facts, and as I do not immediately recollect having any where read of a case of twin-births similar to the one I have here detailed, or met with during a period of thirty years, I send it for insertion in your useful Journal.

Ann Binder, a young woman about sixteen, being in her first pregnancy, was taken in labour on Sunday afternoon, the 24th of July, 1809. She was attended by a very experienced midwife, but not being able to accomplish the delivery, she sent for me about seven o'clock; when I arrived, she informed me it was a twin case, and the first had presented by the feet. On passing an examination, I discovered that the body of the child had protruded through the external orifice, with the arms down on each side; the funis umbilicalis, extremities, and part of the parieties of the abdomen, were in a state of putrefaction; therefore I concluded this child, for some considerable time, had lost its existence; the abdomen was towards the pubis of the patient, and was so completely pinioned or impacted in this way, in the external orifice, which was very contracted and rigid, that it was not without some considerable time and trouble I could extract the shoulders; and with much difficulty, with all the care, attention, and circumspection possible, prevented a laceration of the perinæum. I naturally concluded that when the shoulders were cleared through the os externum, the head would have immediately followed; but still finding a very great obstacle in its descent, and fearing if too much force were applied, I might have separated the head from the body; and well knowing what must follow in such a case, that it must have been left in the cavity of the uterus, had it been so separated, and must have been extracted after the birth birth of the second, as in embryotomy, with the long scissars and crotchet; and as it occurred, from the child being some time in a putrefactive state, the neck was in part dislocated with the little exertion I had used on this occasion, I desisted from any further attempts, till I was fully convinced of the cause that impeded the delivery. On passing another examination, I found the head of the second child had passed that of the first, into the inferior aperture of the pelvis, with the vertex towards the pubis, and the face towards the sacrum, the head of the first still resting on the symphisis of the ossa pubis; in such a situation, as there was no deficiency of uterine action, or want of labour pains throughout the whole process, I suffered the labour to proceed as in cases where there are only one, and about nine o'clock in the evening she was delivered of the second. After the labour had been so far concluded, the head of the first passed downwards with only a single pain, and was protruded through the external orifice so rapidly, and with so much violence against my left hand, that I could scarcely maintain it to support the perinæum again from laceration.

The united placentæ were afterwards extracted in the usual way about twenty minutes or half an hour afterwards, although the funis umbilicalis of the first had been worn through nearly towards its insertion in the parenchymatous substance of the placenta, in the head of the second passing through the external orifice. They were twin girls, and both born dead; the patient recovered from the parturient state in less than three weeks.

From the concurring circumstances in the above recited case, it would naturally appear that the length of time which had elapsed in endeavouring to extract the body and shoulder of the first child, gave the second the opportunity of descending down and passing that of the first into the lower aperture of the pelvis, and from there being no laxity of uterine action pressing on its contents, quaque versa, there was no possibility of returning the head of the second above the superior aperture of the pelvis, could it have been wished for, but was obliged to submit to the evil then existing, rather than incur the risk of doing far greater mischief to the patient by injuring the soft parts, or what would have been still worse, possibly of rupturing the uterus, or of leaving the head behind, either of which every prudent and cautious accoucheur would rather endeavour to avoid.

Another remarkable circumstance attending this case was, that, in addition to the head of the second child pressing upon the perinæum, expecting on the return of every pain its momentary exit, the neck of the first being so completely wedged wedged in the os externum, there should be no laceration of the perinæum.

I am, Gentlemen, your's, &c.
H. G. CLOUGH, M. D.

